# Extravascular lung water, B-type natriuretic peptide and blood volume contraction for diagnosing weaning-induced pulmonary edema

**DOI:** 10.1186/cc12033

**Published:** 2013-03-19

**Authors:** M Dres, JL Teboul, N Anguel, C Richard, X Monnet

**Affiliations:** 1 Service de réanimation médicale, Hôpital de Bicêtre, Le Kremlin Bicêtre, France

## Introduction

We compared three different methods for diagnosing pulmonary edema induced by weaning from mechanical ventilation: the increase in extravascular lung water index (EVLWI), the increase in B-type natriuretic peptide (BNP) and blood volume contraction, reflected by increases in plasma protein and in hemoglobin concentrations, all observed during a spontaneous breathing trial (SBT).

## Methods

We included 12 difficult-to-wean patients (22 recordings). Before and at the end of a SBT (T tube), we measured pulmonary occlusion arterial pressure (PAOP), EVLWI (PiCCO device), BNP, plasma protein and hemoglobin concentrations. Weaning-induced pulmonary edema was confirmed if a clinical intolerance to SBT was associated with an increase of PAOP >18 mmHg at the end of SBT.

## Results

A weaning-induced pulmonary edema was diagnosed in 12 instances (PAOP significantly increased from 15.6 ±3 0.6 to 25.83 ± 0.9 in these cases). EVLWI, BNP, plasma protein and hemoglobin concentrations significantly increased in these instances (28.3 ± 5.7%, 20.2 ± 7.8%, 9.6 ± 0.8% and 9.3 ± 1.3%, respectively) while they did not significantly changed in cases without weaning-induced pulmonary edema. The increase of EVLWI ≥8.5% (+1.53 ml/kg), an increase in BNP ≥6.7% (+23 pg/ml), an increase in plasma protein concentration ≥5% and in hemoglobin concentration ≥5% exhibited good areas under the ROC curves to predict weaning-induced pulmonary edema (0.97 ± 0.03, 0.80 ± 0.11, 1.0 ± 0.00 and 0.92 ± 0.05, respectively). These areas under the ROC curves were not statistically different. The baseline values of EVLWI, BNP, plasma protein and hemoglobin concentrations did not predict weaning-induced pulmonary edema.

## Conclusion

The increases in EVLWI, in plasma protein and hemoglobin concentration and in BNP are valuable alternatives to the pulmonary artery catheter for diagnosing weaning-induced pulmonary edema.

